# Development and validation of the academic stressors scale and its short version

**DOI:** 10.3389/fpsyg.2026.1790872

**Published:** 2026-05-21

**Authors:** Guogang Xin, Shengqin Yang, Ruobing Wang, Jipeng Wang, Shuaishuai Mi

**Affiliations:** 1 School of Government, Beijing Normal University, Beijing, China; 2 Faculty of Education, University of Macau, Taipa, Macao SAR, China; 3 International Institute of Chinese Studies, Beijing Foreign Studies University, Beijing, China; 4 School of Chemistry Chemical Engineering and Materials, Shandong Normal University, Jinan, China

**Keywords:** academic stress, high school student, item response theory, network analysis, scale development

## Abstract

**Introduction:**

This study developed and validated the Academic Stressors Scale (ASS) to measure sources of academic stress among secondary school students. The scale was developed based on a comprehensive theoretical framework that categorizes academic stressors into four key dimensions: parent, self, peer, and teacher stressors.

**Methods:**

A cross-sectional survey was conducted with 11,704 high school students across 27 provinces in China.

**Results:**

The scale was developed within a theory-driven four-factor framework encompassing parent-, self-, peer-, and teacher-related stressors. Its factor structure was supported by exploratory factor analysis and confirmatory factor analysis. In addition, measurement invariance testing demonstrated that the scale functioned equivalently across gender and regional groups. The scale demonstrated good internal consistency and criterion-related validity, with academic stress correlated to anxiety positively and hope negatively. Additionally, its short version with six core items was refined using network analysis and item response theory to enhance efficiency while maintaining measurement precision.

**Discussion:**

The results indicate that the ASS is a reliable and valid instrument for assessing academic stressors in secondary school students. It serves as a valuable tool for psychological assessment, educational interventions, and future research.

## 1 Introduction

Academic stress has strong impact on adolescents' development, profoundly impacting their mental health, academic performance, and social adaptability. During secondary school years, it is widely recognized as a major source of stress for adolescents. The level of stress experienced by students often exceeds their capacity to cope. Excessive stress is associated with psychological problems such as anxiety, depression and suicide attempts (Steare et al., 2023), as well as physical health problems like insomnia (Zhu et al., 2021). Furthermore, such stress can adversely affect adolescents' social relationships, leading to strained peer interactions and family conflicts (Jiang et al., 2022). The overload may have a negative impact on adolescents' long-term development. To effectively manage students' academic stress, it is necessary to accurately identify its sources in order to promote adolescents' mental health and academic success.

### 1.1 Research gaps

Scholars have developed various academic stress scales to measure and study academic stress. Examples include the Academic Expectations Stress Inventory (AESI; Ang and Huan, 2006), the Educational Stress Scale for Adolescents (ESSA; Sun et al., 2011), the Iranian Adolescent Academic Stress Questionnaire (IAASQ; Hosseinkhani et al., 2020), and the Questionnaire on Academic Stress in Secondary Education (QASSE; García-Ros et al., 2018). Among these, AESI and ESSA exhibit high reliability and validity and have been validated across different cultural contexts, making them widely used (Ang et al., 2009; Moustaka et al., 2023; Nguyen, 2015; Tan and Yates, 2011; Truc et al., 2015).

Although AESI and ESSA focus on the students' experiences about academic stress, they differ in their specific emphasis. AESI is designed to assess their stressful events caused by academic expectations, particularly within Asian cultural contexts. Researchers identified two key indicators—parent/teacher expectations and self-expectations—by examining students' stressful events regarding expectations from parents, teachers, and themselves (Ang and Huan, 2006). However, its focus on expectation-related stress alone means it lacks a comprehensive assessment of other important stressful events, such as academic workload, learning burnout, and peer stress, which may limit its applicability.

In contrast, ESSA provides a multidimensional assessment of adolescent academic stress (Sun et al., 2011). The final scale includes five indicators: learning pressure, academic workload, concerns about academic performance, self-expectations, and feelings of frustration. Despite its contributions to academic stress measurement, the indicators of ESSA lack a clear theoretical framework. This may reduce its content and construct validity, as the five indicators were derived from data-driven exploratory factor analysis and are not clearly defined.

Previous scales blurred the distinction between stressful events, perceived stress and stressed behavior in item design. According to the cognitive-phenomenological-transactional (CPT) theory (Lazarus, 1993), stressors refers to the stimuli in activities that induce stress. The evaluation of stressed behavior would lead to outward expressions of stress. However, the way stress manifests depends on internal appraisal, coping strategies, and social support, which explains why the correlation between stressed behavior and stress perception is below 0.4 (Lin, 2014). This suggests that simply asking how a student feel and behave stressed is inappropriate. For example, asking students whether they agree with the statement “I usually cannot sleep because of…” (Ang et al., 2007) equates the externalized and internalized problems caused by academic stress with stress itself. Instead, stressors are more suitable as scale item statements.

Additionally, some previous scales mistakenly used the event frequency and agreement as the Likert response options, deviating from the CPT framework. For example, AESI used 1-Never to 5-Always (Ang et al., 2007). However, the event frequency does not directly reflect students' evaluation about the events, some students may experience the event without feeling stressed. And ESSA used 1-Strongly disagree to 5-Strongly agree. The response options are confusing because the “disagree” category can be interpreted in two ways. It may indicate that students feel happy or delighted when experiencing the event, or that they experience no stress. And the options did not consider what the students should select when students did not experience the events (Sun et al., 2011). As an alternative design, the description about how students feel about their perceived academic stress is better suited for response options.

Lastly, previous academic stress scales lack normative data and short-form versions, which limits their practical application in assessment, prevention, and intervention efforts. The absence of norms means that researchers and educators cannot determine whether a student's stress level is at the normal range, making it difficult to assess the severity of stress scores (American Educational Research Association, 2014). However, this issue has been largely overlooked in prior research. Additionally, the length of full-length academic stress scales may reduce assessment efficiency, especially in large-scale screening or rapid evaluation contexts (Smith et al., 2000). To improve efficiency and reduce response burden, it is essential to develop short-form versions that maintain validity and reliability while being more practical for large-scale surveys, quick screenings, and intervention evaluations.

### 1.2 Research aims

The purpose of the study is to develop an academic stressors scale that can solve the problems mentioned above. It seeks to make several key improvements, including:

(1) To improve item design, this study reviewed and refined items from previous scales. Items describing stressful events were retained, whereas those reflecting stress behaviors were removed to enhance theoretical consistency. In addition, to address limitations in prior response formats, the new scale adopts stress perception as the response option, which is considered more precise than frequency-based or agree–disagree formats.
(2) For solving the lack of construct theory, the study conducted such a process to develop the scale: A two-dimensional theory about academic stress construct is developed based on the analysis upon provious research, which highlight the characteristics of event topics and related people. And the remained items were categorized into cells in this two-dimensional table that aligns with the theoretical framework. Then the most suitable item for each cell was selected from the item pool according to the consensus of experts. Thus, the new scale is theory-based and of good content validity. And it allows a rigorous examination on structural validity.
(3) A cross-sectional questionnaire survey is conducted to validate the scale's structural validity, measurement invariance, criterion-related validity, and reliability. It also establishes normative data and develops a short-form version for practical use. Thus, the scale would have comprehensive evidence for its quality and adaptability.
(4) A short-form version scale was developed for practical use. The critical items were selected from the full-length scale according to the results of network analysis and item response theory (IRT). From a network perspective, academic stress is viewed as a system of interacting stressors, with nodes representing stressors and edges indicating their relationships (Li et al., 2025). Network analysis identifies high-centrality items (core stressors) that strongly influence the network (Liang et al., 2023). These items have been shown to outperform traditional short-form scales and were therefore prioritized (Liang et al., 2023). Furthermore, IRT was used to further refine the scale. The Item Information Curve (IIC) assesses how much information each item provides across trait levels (Embretson and Reise, 2013). Items with higher information values provide greater precision and were retained. IRT was then used to validate these items and ensure the precision of the short-form scale.

## 2 Methods

### 2.1 Participants and procedure

Two rounds of questionnaire data collection were conducted: the first round was a pilot survey, and the second round was the formal survey.

The pilot survey was used to examine construct validity by exploratory factor analysis. Cluster sampling was employed, and a survey was conducted in April 2023 among 445 senior high school students from a high-performance high school in a town in Henan Province. A total of 403 valid samples were obtained (unanswered or regularly answered samples were considered invalid and removed).

The formal survey employs convenience sampling in 2025 spring, with the test embedded in an application provided by a company, offering pre-exam mental health diagnostic services to students in partner schools. The participants are senior high school students from 27 provinces across China, surveyed 1 month before their college entrance examination. They can choose to click a button in the app to use the mental health diagnostic service and expose to the scale, and they are free to exit without influencing other functions of the app. The app would notice them that their response would be used in the research and explain the purpose of the research. Participants need to click the informed consent button, those who reject to click the informed consent button can still use the scale but their response will not be uploaded in the database. Informed consent was obtained from all subjects. All data were collected anonymously and contain no identifiable human information. The study was approved by the Shandong Normal University's Ethics Committee (Approval No. 20250305).

The sample consists of 14,186 senior high school students. Responses failing the polygraph items or exhibiting abnormal test durations were considered invalid, resulting in 11,704 valid samples. Among them, 4,920 were female (42.0%) and 6,784 were male (58.0%). Based on geographical distribution, the sample was categorized into four regions in China: Eastern (2,898, 24.8%), Central (3,880, 33.2%), Western (3,524, 30.1%), and Northeastern (1,402, 12.0%), in which students may have different response modes due to their different stage of economic development and different culture especially in education.

### 2.2 Measures

#### 2.2.1 Academic stress

Academic stressors scale consists of 21 items measured on a five-point Likert scale. The process and detailed information of developing this scale has been described in Sections 1.2 and 3.1. Each statement of item represents an academic stress event, requiring participants to rate whether the event caused them stress. The scale comprises four stressors representing different social sources (parents, teachers, peers, and self) in stressful events: items 1–6 represent the parent stressor, items 7–11 represent the self-imposed stressor, items 12–16 represent the peer stressor, and items 17–21 represent the teacher stressor. Items in each indicator represent different event topics. Contents of item statements can be found in Table 1. Following previous scales, the instruction for the new scale reads: “In the past 2 weeks, which of the following have caused you stressed?” The five response options are scored from 0 to 4. The response options include “Did not cause me stressed,” “Slight stressed,” “Moderate stressed,” “Considerable stressed,” and “Severe stressed that I cannot bear.”

**Table 1:** Item attribute table.

Event types	Social relationships			
	Parent	Self	Peer	Teacher
Long-term academic expectations	T1. Parents often nag at me, wanting me to be a great person.	T7. My worries about the future.	×	T17. Teacher has set expectations for my future development.
Learning task	T2. In life, my parents are too strict with me.	T8. I don't have time for anything other than studying, my interests are not satisfied.	×	T18. Teacher assigns more or more difficult learning tasks.
Short-term academic expectations	T3. Parents will be annoyed if their test scores are not good.	T9. I am not satisfied with my learning results.	T12. Classmates often talk about exams.	×
Setbacks	T4. Parents blame me.	T10. I can't achieve the goals I set for myself.	T13. When I failed, others mocked me.	T19. Teacher criticized me.
Interpersonal relationship	T5. Tension with parents.	×	T14. When I am troubled, I have no close friends to talk to. T15. I often clash with my peers.	T20. Teacher didn't care about me and almost ignored my existence.
Academic competition	T6. Parents always compare me to other people's children.	T11. I hope to win the respect of others through my grades.	T16. Want to exceed a peer's academic performance.	T21. Teacher always compares me to other peers.

#### 2.2.2 Anxiety and hope

Anxiety is assessed using the short version of the Generalized Anxiety Disorder Scale, which includes two items (Donker et al., 2011). Responses are rated based on frequency, ranging from 0-never to 3-almost every day. A total score of 3 or higher indicates a risk of generalized anxiety disorder. In this study, the Cronbach's α for this scale is 0.858.

Hope is measured using the second dimension of the Adult Dispositional Hope Scale (Chen et al., 2009), which consists of four items scored on a four-point scale. Higher scores indicate stronger hopeful emotions. The Cronbach's α for this scale in the present study is 0.718.

### 2.3 Statistical analyses

The research conducted a series of statistical analyses to provide evidence for reaching the research aims. The content validity indexes are used to examine the content validity of ASS. The significancy to correlation coefficient between ASS and anxiety/hope provided evidence for criterion-related validity. The results of EFA and CFA examined its structural validity. The measurement invariance tests examined the equity of measurement and supplement the information about its structural validity. The Cronbach's α coefficient examined the internal consistency reliability of ASS. The ROC helped to construct the norms of academic stress by providing cutoff ranges based on the risk of anxiety disorder. The network analysis and IRT are used to select items for short-from.

JASP 0.19, Mplus 8.3, and R 4.0.4 were used to analyze the data. JASP 0.19 were used for exploratory factor analysis, Cronbach's α coefficient, correlation coefficient, and receiver operating characteristic (ROC) analysis. Mplus 8.3 was used for confirmatory factor analysis and measurement invariance testing. And R 4.0.4 was used to conduct network analysis and IRT analysis.

In terms of criterion-related validity, we validated it by examining the correlation between the Academic Stress Scale and levels of anxiety and hope. According to the control-value theory (Pekrun, 2006), academic emotions arise from students' appraisals of control and value. When students perceive high control over academic activities and attach high subjective value to them, they experience positive emotions; such emotions facilitate effective self-regulation and task engagement, thereby reducing perceived academic stress. In contrast, when control is low but value remains high, negative emotions emerge, which intensify stress by undermining coping resources. Moreover, high-arousal emotions are more directly tied to ongoing or anticipated academic activities and outcomes, producing stronger and more immediate effects on motivational and cognitive processes than low-arousal emotions, which primarily reflect settled appraisals and thus correlate less strongly with acute stress (Pekrun, 2006). Pekrun (2006) argued that, hope arises from students' perceived control on high subjective value achievement. Internally endorsed values combined with perceived control generate higher intensity of hope than externally imposed values do. According to existing research findings (Gao, 2022): (1) Academic stress is positively correlated with negative emotions and negatively correlated with positive emotions. (2) Academic stress has a stronger correlation with high-arousal emotions than with low-arousal emotions. (3) Self-imposed stress has a stronger correlation with positive emotions than other dimensions of academic stress. In this study, anxiety represents a high-arousal negative emotion, while hope represents a low-arousal positive emotion. Based on this, the following hypotheses are proposed to further validate the validity of the Academic Stress Sources Scale: (1) Academic stress and its dimensions are negatively correlated with anxiety and positively correlated with hope. (2) The correlation coefficient between total academic stress and anxiety is greater than that between academic stress and hope. (3) The correlation coefficient between self-imposed stress and hope is greater than that of other dimensions.

R 4.0.4 was used to estimate the corresponding network, following standard guidelines from previous research (Epskamp et al., 2018). First, the R package bootnet was used to estimate the network model of academic stress sources and to estimate and visualize the node network. Second, centrality indicators (expected influence) were used to assess the role of each node in the network (Robinaugh et al., 2016). Items ranked highest in centrality values were selected to form a simplified version of the scale. Then, accuracy analysis was conducted on edge estimation and centrality estimation. First, the accuracy of edge estimation was evaluated using the 95% confidence interval of bootstrapped edge weights, where a smaller confidence interval indicates more accurate edge estimation. Second, a subset bootstrap procedure was used to remove a certain proportion of participants and re-estimate node centrality. When the correlation between the new centrality estimates and the original centrality indicators reached 0.7, the proportion of removed participants was defined as the Centrality Stability Coefficient (CS-coefficient). A CS coefficient greater than 0.25 indicates acceptable stability, while a coefficient greater than 0.50 indicates good stability.

The mirt package in R 4.0.4 was used to plot item information curves for all items, determining which items provide the most information within the θ = (-2.5, +2.5) range, since more than 95% participants lie in the range. Items were then ranked based on their maximum information value. Based on this ranking, recommendations were made: priority was given to retaining items with high information values, while the retention of moderately informative items was determined based on additional evidence. Items with low information values were removed.

To ensure that the data satisfy the prerequisite assumptions of analysis tools (EFA, CFA, ROC, IRT), a series of tests were conducted, including: (1) the sample sizes for EFA/CFA/IRT exceeded 10 times the number of items (the sample size used for EFA in this study was 403, and the sample size used for CFA/IRT was 14,186); (2) the items used in the CFA exhibited linear correlations (SRMR = 0.046, indicating that the average absolute residual was less than 0.05 between the model-implied and the observed correlation matrix; the average Pearson correlation coefficient among items was 0.179, and all were significance, indicating that the linear model was an effective approximation for CFA); (3) the data used for EFA/CFA showed no multicollinearity (the multicollinearity diagnostics showed a minimum tolerance = 0.393 > 0.1 and a maximum VIF = 2.545 < 10, indicating no serious multicollinearity issues); (4) the data used for CFA did not satisfy multivariate normality (standardized multivariate skewness coefficient = 17.38, *p* < 0.001; standardized multivariate kurtosis coefficient = 538.88, *p* < 0.001, failing to meet the assumption of multivariate normality; therefore, MLMV estimation was adopted in the CFA for robust estimation); and (5) local independence (Yen's Q3 values between all items in the IRT data were all less than 0.2, indicating no serious local dependence issues).

## 3 Results

### 3.1 Development of ASS

#### 3.1.1 Item development

This study conceptualized stress sources across four domains (parents, teachers, peers, and self) based on the bioecological systems theory. The academic stress come from the external stimuli to the outside environment (Lazarus, 1993). The bioecological systems theory further argues that, individuals live in a nested environment, and they interact with the microsystem frequently and directly (Bronfenbrenner, 1977). Practically, it is realistic to capture students' recent and frequent interaction in family and school (Cusato, 2022; Paola, 2006) by incorporating the indicators of parents, teachers, and peers, since they are the significant others (Engle and Szyperski, 1965; Kemper, 1966) and major reference group (Kelley, 1952) that have the most influence on high school students. Furthermore, there have been several instruments including these elements, showing that parents (Ang and Huan, 2006; Rao, 2008), teachers (Ang and Huan, 2006; Hosseinkhani et al., 2020) and peers (Boyle, 2020; Xu et al., 2010) could be used in the academic stress construct.

However, it would be difficult to capture those the long-term and subtle influence from less frequent and indirect interactions from neighborhoods, culture, customs, and media (Cusato, 2022). Students gradually internalize academic norms through prolonged exposure to such influence (Ryan, 1985), and their self-imposed academic stress could be an alternative solution for incorporating the other levels of students' bioecological system (Paola, 2006). There has been evidence that self should be an element of academic stress scale (Chen, 2004; García-Ros et al., 2018). In practice, such a four-element framework has been adopted in previous academic stress scale (Xu et al., 2010).

From the other dimension, different people may trigger similar academic stressor events, thus such events can be categorized in the same event topic. Scholars generally agree that expectations for academic achievement are a crucial type of academic stress event, a conclusion applicable across different cultures and educational systems (Ang et al., 2007). Specifically, academic expectations can be further divided into expectations for long-term academic achievement and expectations for the completion of short-term academic tasks (Ge, 2008). Additionally, academic stress often arises from intense academic competition, academic setbacks, excessive or overly difficult learning tasks, and interpersonal tensions triggered by academic matters (Chen, 2004; Wang, 2008; Xu et al., 2010). Based on these studies, each stressor related to significant others may encompass six categories of event topics: long-term academic expectations, short-term academic expectations, academic competition, setbacks, learning task, and interpersonal relationship.

Item attribute table (Table 1) was constructed with the four stress sources as rows and the six event topics as columns. Additionally, a literature review identified four rigorously developed scales with content consistent with the CPT theory, suitable for the Chinese cultural and educational environment, and applicable to secondary school students (Chen, 2004; Ge, 2008; Wang, 2008; Xu et al., 2010). The research team met to discuss the meaning of each cell in the table. Based on this discussion, the items from the four scales were categorized and entered into the item attribute table. Any disagreements were further discussed until consensus was reached. Items from these four scales were placed into the item attribute table, excluding those beyond the construct's scope (e.g., items related to the physical environment such as “lack of a quiet study environment”) or items that could fit into multiple categories (e.g., “parents or teachers believe that only good grades define a good student”). Four gaps in the table remained without corresponding items, possibly due to specific people triggering only certain topics of stress events.

Among the 24 positions in the item attribute table, one item was selected for each position based on its broad applicability. This selection process was carried out by a team of three researchers. Consensus was reached after several rounds of discussion. For instance, within the academic setbacks dimension of the teacher stress source, potential items included “teacher criticizes me” and “teacher severely punishes or humiliates me.” The former was chosen because it encompasses the latter and occurs more frequently in reality. A total of 21 items were selected to form a new scale (with two items under peer-interpersonal relationship stress to balance the number of items per row and column).

#### 3.1.2 Content validity

Seven experts in education and psychology were recruited to evaluate the quality of the items in terms of relevance, comprehensiveness, and comprehensibility. They completed an evaluation form and rating scale to determine whether each item was unrelated, weakly related, moderately related, or highly related. The content validity indices include the Item-Level Content Validity Index (I-CVI) and the Scale-Level Content Validity Index (S-CVI). The I-CVI is calculated as the proportion of experts rating an item as highly relevant or very relevant, while K^*^ adjusts this value for chance agreement. The S-CVI is obtained by averaging the I-CVI values of all items. Typically, an I-CVI greater than 0.5 and an S-CVI greater than 0.9 are required; K^*^ values are classified as fair (0.40–0.59), good (0.60–0.74), or excellent (>0.74; Lynn, 1986).

The I-CVI and K^*^ results are presented in Table 2, with S-CVI = 0.939. Evaluations from seven experts indicate that all items have an I-CVI above 0.5, and the overall S-CVI exceeds 0.9. The majority of items have a K^*^ value classified as excellent, while three items are rated as good. Therefore, all content validity indices for the scale meet psychometric standards, confirming that the item content aligns well with the intended constructs.

**Table 2:** Expert evaluation table.

Item	Unrelated	Weakly related	Moderately related	Highly related	I-CVI	*K* ^*^
1	0	0	2	5	1.00	1.00
2	0	0	4	3	1.00	1.00
3	0	0	1	6	1.00	1.00
4	0	0	2	5	1.00	1.00
5	0	1	3	3	0.86	0.85
6	0	0	4	3	1.00	1.00
7	0	0	2	5	1.00	1.00
8	0	0	4	3	1.00	1.00
9	0	0	2	5	1.00	1.00
10	0	0	4	3	1.00	1.00
11	0	0	3	4	1.00	1.00
12	0	0	4	3	1.00	1.00
13	0	0	2	5	1.00	1.00
14	1	1	2	3	0.71	0.65
15	1	1	2	3	0.71	0.65
16	0	0	3	4	1.00	1.00
17	0	1	3	3	0.86	0.85
18	0	0	3	4	1.00	1.00
19	0	2	2	3	0.71	0.65
20	0	1	4	2	0.86	0.85
21	0	0	3	4	1.00	1.00

### 3.2 Reliability and validity of ASS (pilot survey)

#### 3.2.1 Reliability

The overall scale has a Cronbach's α of 0.880, in which the reliability coefficients for the parent, self-imposed, peer, and teacher are 0.838, 0.792, 0.728, and 0.817, respectively. A reliability coefficient greater than 0.9 indicates good excellent reliability, ≥0.8 Good, ≥0.7 Acceptable, ≥0.6 Questionable, ≥0.5 Poor, and ≤ 0.5 Unacceptable (George and Mallery, 2003). For subscales, the reliability threshold is typically lowered by 0.1 (Hair et al., 2019). The results indicate that the overall scale and the subscales demonstrate good internal consistency.

#### 3.2.2 Exploratory factor analysis

The Kaiser-Meyer-Olkin (KMO) measure of sampling adequacy is 0.875 (>0.6), indicating that the data are suitable for factor analysis. Bartlett's test of sphericity yields χ^2^ = 3,311.716, *df* = 210, *p* < 0.001, confirming that the correlation matrix is not an identity matrix, further supporting the appropriateness of factor analysis. The scree plot indicates a clear elbow at the fourth factor, where the eigenvalues begin to level off (Supplementary Figure S1). In addition, the first four factors have eigenvalues greater than 1, while subsequent factors fall below this threshold. These results jointly support the retention of four factors. Four factors were extracted, with items 1–6 loading onto the first factor, items 7–11 and 16 onto the second factor, items 12–15 onto the third factor, and items 17–21 onto the fourth factor, as shown in Table 3. The results indicate that the item loadings align with theoretical assumptions, though item 16 exhibits cross-loading across factors.

**Table 3:** Exploratory factor analysis.

Item	Parent	Self	Peer	Teacher	MSA
1	0.702				0.863
2	0.798				0.880
3	0.745				0.908
4	0.745				0.872
5	0.692				0.866
6	0.631				0.909
7		0.769			0.878
8		0.537			0.895
9		0.810			0.840
10		0.809			0.818
11		0.669			0.879
12			0.419		0.859
13			0.650		0.897
14			0.689		0.877
15			0.709		0.881
16		0.501	0.263		0.932
17				0.574	0.896
18				0.690	0.838
19				0.787	0.880
20				0.702	0.864
21				0.807	0.865

### 3.3 Reliability and validity of ASS (formal survey)

#### 3.3.1 Reliability

The overall scale has a Cronbach's α of 0.820, in which the reliability coefficients for the parent, self-imposed, peer, and teacher are 0.846, 0.703, 0.578, and 0.536, respectively. The results indicate that the overall scale demonstrates good internal consistency. The parent and self-imposed stress indicators exhibit relatively high reliability, while the peer and teacher indicators have acceptable reliability.

#### 3.3.2 Confirmatory factor analysis

**Figure 1:**
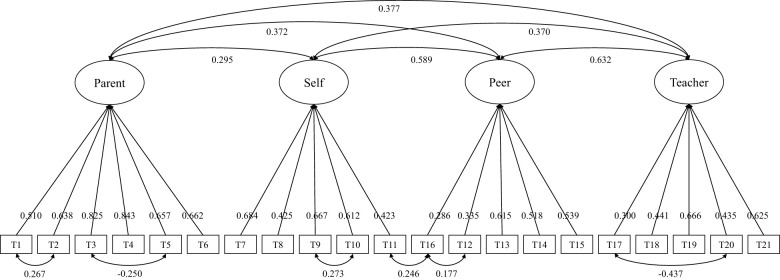
Confirmative factor analysis structure and load.

Confirmatory factor analysis (CFA) was conducted on the data, using a baseline model in which all items loaded onto a single factor and a theoretical model constructed with four stress source dimensions as factors. The chi-square test results, information criteria (AIC, BIC), and fit indices (RMSEA, CFI, TLI, SRMR) for both models are presented in Table 4. The theoretical model's structure and factor loadings are illustrated in Figure 1. In the CFA analysis, an RMSEA between 0.05 and 0.08 indicates a good fit, while CFI and TLI values above 0.9 and an SRMR below 0.08 indicate that the model meets the fit criteria. The information criteria values for the theoretical model are lower than those for the baseline model, suggesting that the theoretical model provides a better fit. The results demonstrate that the formal test data meet the fit standards, with the theoretical model achieving a good fit. Comparing the exploratory factor analysis (EFA) and CFA results, the statistical model that best fits the data aligns with the theoretical construct, confirming the structural validity of the scale.

**Table 4:** Comparison of statistical indicators between baseline model and theoretical model.

Indicators	Baseline model	Theoretical model
χ^2^, *df*	29,072.682, 189	5,477.311, 177
AIC	719,424.983	695,853.613
BIC	719,889.137	696,406.176
RMSEA	0.114	0.051
CFI	0.538	0.915
TLI	0.488	0.900
SRMR	0.103	0.046

#### 3.3.3 Measurement invariance tests

The measurement invariance analysis was conducted to examine the structural stability of ASS across students of different genders and regions (Eastern, Central, Western, and Northeastern).

The measurement invariance test across genders is presented in Table 5. The results of the separate sample confirmatory factor analysis indicate that the model structures are well-fitted for both male and female samples (Female model: CFI = 0.927, TLI = 0.915, RMSEA = 0.051; Male model: CFA = 0.918, TLI = 0.904, RMSEA = 0.051). In the measurement invariance test, compared to the configural invariance model, the metric invariance model did not show a significant deterioration in fit (ΔCFI = 0.000, ΔRMSEA = −0.002, Δχ^2^ = 52.235, *p* < 0.01). Similarly, compared to the metric invariance model, the scalar invariance model did not show a significant deterioration in fit (ΔCFI = −0.012, ΔRMSEA = +0.002, Δχ^2^ = 657.892, *p* < 0.001). Furthermore, compared to the scalar invariance model, the residual variance invariance model also did not show a significant deterioration in fit (ΔCFI = −0.006, ΔRMSEA = 0.002, Δχ^2^ = 389.072, *p* < 0.001). These results indicate that the measurement invariance across genders was supported.

**Table 5:** Measurement invariance test results by gender.

Model	χ^2^	*df*	CFI	TLI	RMSEA	Δχ^2^	ΔCFI	ΔRMSEA
All	4,497.172	146	0.923	0.910	0.050	–	–	–
Female	2,027.221	146	0.927	0.915	0.051			
Male	2,706.135	146	0.918	0.904	0.051	–	–	–
Configural	4,733.355	292	0.922	0.909	0.051	–	−0.001	+0.001
Metric	4,785.590	315	0.922	0.915	0.049	52.235^**^	0.000	−0.002
Scalar	5,443.482	334	0.910	0.908	0.051	657.892^***^	−0.012	+0.002
Error	5,832.554	355	0.904	0.907	0.051	389.072^***^	−0.006	0.000

χ^2^, Chi-square; df, degrees of freedom; CFI, comparative fit index; TLI, Tucker-Lewis Index; RMSEA, root-mean-square error of approximation; Δχ^2^, ΔCFI ΔTLI and ΔRMSEA, change in χ^2^, CFI/TLI, RMSEA values compared to the preceding model. ^**^p < 0.01, ^***^p < 0.001.

The measurement invariance test across regions is presented in Table 6. The results of the separate sample confirmatory factor analysis indicate that the model structures are well-fitted for samples from the Eastern, Central, Western, and Northeastern regions (Eastern model: CFI = 0.923, TLI = 0.909, RMSEA = 0.051; Central model: CFA = 0.928, TLI = 0.916, RMSEA = 0.048; Western model: CFI = 0.918, TLI = 0.904, RMSEA = 0.053; Northeastern model: CFI = 0.914, TLI = 0.900, RMSEA = 0.055). In the measurement invariance test, compared to the configural invariance model, the metric invariance model did not show a significant deterioration in fit (ΔCFI = −0.000, ΔRMSEA = +0.001, Δχ^2^ = 120.052, *p* < 0.01). Similarly, compared to the metric invariance model, the scalar invariance model did not show a significant deterioration in fit (ΔCFI = −0.002, ΔRMSEA = −0.001, Δχ^2^ = 170.287, *p* < 0.001). Furthermore, compared to the scalar invariance model, the residual variance invariance model also did not show a significant deterioration in fit (ΔCFI = −0.002, ΔRMSEA = −0.001, Δχ^2^ = 186.389, *p* < 0.001). These results indicate that the measurement invariance across different regions was supported.

**Table 6:** Measurement invariance test results in different regions.

Model	χ^2^	*df*	CFI	TLI	RMSEA	Δχ^2^	ΔCFI	ΔRMSEA
All	4,497.172	146	0.923	0.910	0.050	–	–	–
East	1,239.337	146	0.923	0.909	0.051	–	–	–
Mid	1,450.559	146	0.928	0.916	0.048	–	–	–
West	1,571.892	146	0.918	0.904	0.053	–	–	–
Northeast	755.546	146	0.914	0.900	0.055	–	–	–
Configural	5,017.335	584	0.922	0.909	0.051	–	−0.001	+0.001
Metric	5,137.387	653	0.921	0.917	0.048	120.052^**^	−0.001	−0.003
Scalar	5,307.674	710	0.919	0.922	0.047	170.287^***^	−0.002	−0.001
Error	5,494.063	773	0.917	0.927	0.046	186.389^***^	−0.002	−0.001

^**^p < 0.01, ^***^p < 0.001.

#### 3.3.4 Criterion-related validity

**Figure 2:**
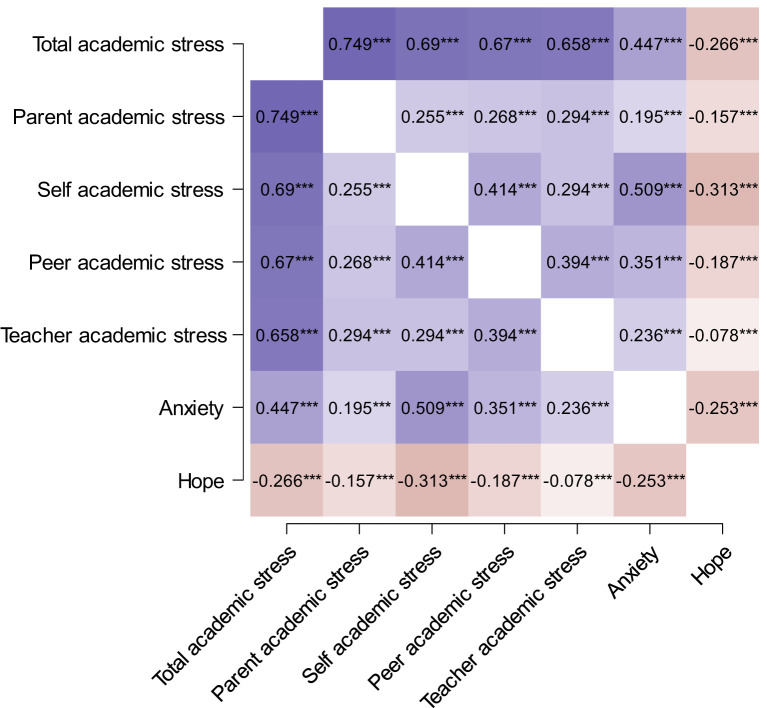
The correlation between the total academic stress score, its dimensions, hope and anxiety. ***p < 0.001.

The correlation coefficients between the total academic stress score, its dimensions, and both hope and anxiety are presented in Figure 2. The results indicate that academic stress and all its dimensions are significantly negatively correlated with anxiety and significantly positively correlated with hope. Further tests were conducted to examine the differences between correlation coefficients (Lenhard and Lenhard, 2014). The results showed that the correlation coefficient between total academic stress and anxiety was significantly greater than that between total academic stress and hope (*z* = 17.719, *p* < 0.001). Additionally, the correlation between hope and self-imposed stress was significantly higher than its correlation with the dimensions of parent, peer, and teacher-related stress (*z* = 14.418, *p* < 0.001; *z* = 13.155, *p* < 0.001; *z* = 12.01, *p* < 0.001).

A structural equation model was constructed with the four dimensions of academic stress as predictors and hope and anxiety as outcome variables (Supplementary Figure S2). The proposed model demonstrated an acceptable overall fit to the data (χ^2^ = 13,346.592, *df* =309, RMSEA = 0.060, CFI = 0.855, TLI = 0.836). The results also showed that self-imposed stress had a stronger predictive effect on hope than other stress.

These criterion-related results align with the theoretical hypotheses, providing further evidence supporting the criterion-related validity of the scale.

### 3.4 ASS norms

According to the frequency histogram plot and Q-Q plot (Supplementary Figures S3, S4), academic stress follows an approximately normal distribution (*M* = 42.14, *SD* = 11.10). It is generally believed that academic stress and academic achievement exhibit an inverted U-shaped relationship, where moderate stress leads to the highest efficiency, while both excessive and insufficient stress result in poorer performance (Ma, 2023). Based on this theory, academic stress should be categorized into three levels: low, moderate, and high. Sensitivity and specificity were determined through receiver operating characteristic (ROC) analysis, using participant classification as the reference. The anxiety disorder (GAD ≥ 3) was used as the criteria. The area under the curve (AUC) and 95% confidence intervals indicated the overall accuracy. The optimal cut-off scores were identified using the highest Youden index (sensitivity + specificity – 1), which represents a balance between sensitivity and specificity (Hajian-Tilaki, 2018). The two optimal cut-off points were 36.5 (AUC = 0.955) and 46.5 (AUC = 0.824). Therefore, the total score ranges were defined as follows: 0–36 points for low stress, 37–46 points for moderate stress, and 47–84 points for excessive stress. The risk of anxiety disorder for high stress group is far higher than the moderate stress group, while the risk of moderate stress group is slightly higher than the low stress group (Supplementary Figure S5).

### 3.5 Development of a short form of ASS

#### 3.5.1 Network analysis

**Figure 3:**
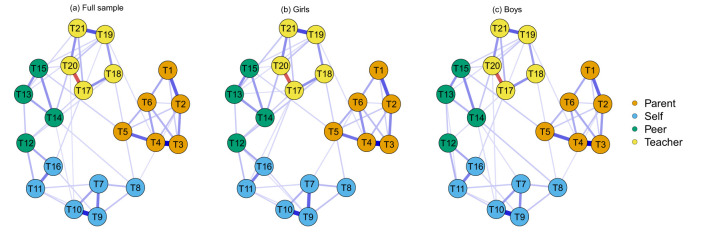
Academic stressor network. Each node represents an academic stressor event, while the edges represent the connections between nodes. The thickness of the edges indicates the strength of the correlation between nodes, with thicker edges representing stronger associations. The color of the edges represents the direction of the correlation, where blue indicates a positive correlation and red indicates a negative correlation. The specific content of each item is detailed in Table 1. Nodes of different colors represent different sources of stress: orange for parent stressors, blue for self, green for peer, and yellow for teacher.

This study employed network analysis to estimate the academic stressor networks for the total sample, male students, and female students. The network consists of 210 edges (21 × (21-1)/2). The total sample, male, and female groups contained 129, 124, and 128 non-zero-weight edges, respectively, with a network density of 0.04 for all three groups. The academic stressor network is presented in Figure 3. Items within the same dimension tended to cluster together. Notably, strong connections were observed between T3 and T4 in the family stressor dimension, T9 and T10 in the self-imposed stressor dimension, and T19 and T21 in the teacher stressor dimension.

**Figure 4:**
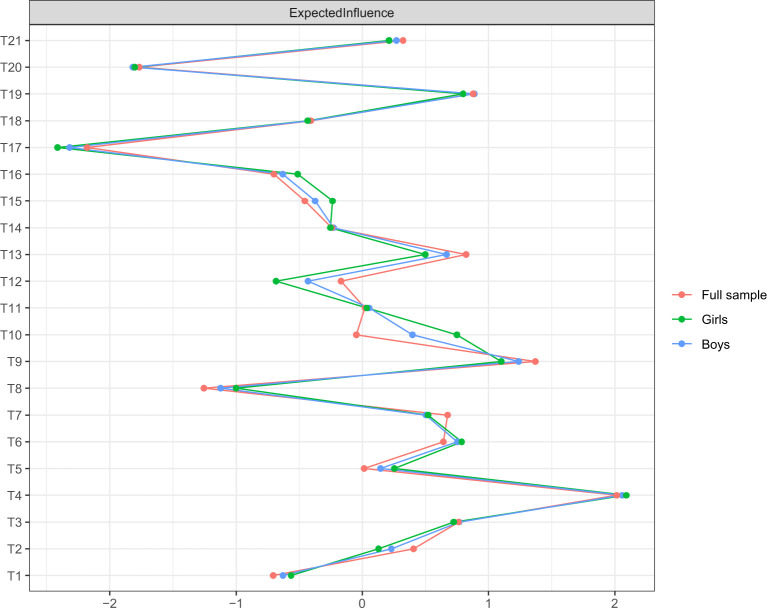
Node strength centrality of each item.

The expected influence centrality of each node is illustrated in Figure 4. In the total sample, T4 (“My parents blame me”) from the family stressor dimension and T9 (“I am dissatisfied with my academic performance”) from the self-imposed stressor dimension exhibited the highest expected influence centrality. These were followed by T19 (“My teacher criticizes me”) from the teacher stressor dimension, T3 (“My parents get angry if I get poor grades”) and T6 (“My parents always compare me with other children”) from the family stressor dimension, and T13 (“When I fail, others mock me”) from the peer stressor dimension. These six nodes had the most substantial impact on the academic stressor network. In both male and female samples, the ranking of the top three nodes (T4, T9, and T19) remained consistent, while the rankings of the remaining nodes varied slightly. However, T3, T6, and T13 remained among the most highly ranked nodes. Therefore, these six items were selected to form the short-form version of the academic stressor scale.

The results of the edge weight bootstrapping procedure are presented in Supplementary Figure S6, indicating that the network's edge estimates are relatively accurate, with minimal overlap between the 95% confidence intervals of edge weights. The correlation stability coefficient (CS-coefficient) for expected influence centrality was estimated using a subset bootstrapping procedure. The results showed that the CS-coefficient for expected influence centrality was 0.75 (>0.50), suggesting that the estimates are sufficiently stable. The results of the subset bootstrapping procedure are presented in Supplementary Figure S7.

#### 3.5.2 IRT

A two-parameter item response model was established based on theoretical assumptions. The model fit indices were as follows: RMSEA = 0.055 (90% CI [0.054, 0.057]), SRMSR = 0.063, TLI = 0.907, and CFI = 0.924. The empirical reliability values were 0.879, 0.785, 0.645, and 0.712. These results indicate that the model is acceptable. In this study, item information curves and maximum information from IRT were used as references to determine the importance of each item within the scale.

The item information curves for each question are shown in Supplementary Figure S8. The selection process was conducted as follows: (1) Items 4, 9, and 19 provided the highest amount of information within specific intervals of the specific trait level θ (academic stress from −2.5 to +2.5) and were irreplaceable; therefore, they were retained. (2) Items 1, 8, 11, 12, 14, 16, 17, 18, and 20 had a maximum information value below 0.5, making them relatively inefficient, and they were prioritized for removal. (3) Items 3, 10, and 21 had relatively high information values, with a maximum information value exceeding 1. Among them, both Item 10 and Item 9 belonged to the self-imposed stress dimension and provided more information for groups with lower levels of academic stress. Since Item 9 provided significantly more information than Item 10, Item 10 was removed. Items 19 and 21 both belonged to the teacher stressor dimension and provided more information for groups with higher academic stress levels. However, Item 19 provided more information than Item 21, so Item 19 was retained, and Item 21 was removed. (4) Items 2, 5, 6, 7, 13, and 15 had moderate information values, with a maximum information value exceeding 0.5, and their retention was considered in relation to theoretical relevance. The information curve distribution of Item 6 better covered individuals at both end of stress levels. For detecting information more sensitively for special groups with high and low stress, item 6 was retained. Additionally, since Item 13 was the only item in the peer stressor dimension with a relatively high information value, it was retained to ensure a comprehensive assessment of different sources of academic stress. Ultimately, Items 3, 4, 6, 9, 13, and 19 were retained to form the simplified academic stressor scale.

#### 3.5.3 Reliability and validity test of the short form of academic stressors scale

The simplified academic stressor scale consists of six items, specifically Items 3, 4, 6, 9, 13, and 19 from the full-scale version, as shown in Table 7. The Cronbach's α for the simplified scale is 0.70, indicating that the internal consistency of the short form has an acceptable reliability. A single-factor structure was tested using confirmatory factor analysis. The results showed RMSEA = 0.063 (90% CI [0.058, 0.069]), CFI = 0.976, and TLI = 0.955, indicating good validity.

**Table 7:** The short form of academic stressors scale.

Item	Description of the item
3	Parents will be annoyed if their test scores are not good.
4	Parents blame me.
6	Parents always compare me to other people's children.
9	I am not satisfied with my learning results.
13	When I failed, others mocked me.
19	Teacher criticized me.

## 4 Discussion

This study developed an Academic Stressor Scale and ultimately established a complete version containing 21 items, categorized into four dimensions: parent stress, self-imposed stress, peer stress, and teacher stress. The scale and its dimensions demonstrated good internal consistency reliability. Additionally, both exploratory factor analysis (EFA) and confirmatory factor analysis (CFA) indicated good structural validity. The measurement invariance test showed consistent measurement structures across different genders and regions, ensuring the scale's applicability across different groups. Furthermore, criterion-related validity analysis revealed that the total score and each dimension were positively correlated with anxiety and negatively correlated with hope, supporting the validity of the scale. ROC analysis was used to determine classification criteria for low, moderate, and high levels of academic stress, providing a practical tool for assessing and intervening in academic stress. To enhance assessment efficiency, the study utilized network analysis and IRT to simplify the scale, ultimately selecting six core items to form the short-form Academic Stressor Scale. The internal consistency reliability and confirmatory factor analysis of the short-form scale indicated good reliability and validity.

The study identified four core dimensions of academic stressors: family, self, teachers, and peers. These dimensions encompass the primary sources of academic stress for secondary school students (Xu et al., 2010), and the selection of scale items ensured alignment with the CPT theory. The core aspect of family stress lies in high parent expectations and strict requirements, as exemplified by the item “My parents get angry if I get poor grades,” which is particularly pronounced in Chinese culture. Many parents directly associate academic performance with future success, placing significant academic stress on students (Ang and Huan, 2006). Self-imposed stress reflects individuals' dissatisfaction with their abilities and achievements, such as “I am dissatisfied with my academic performance,” which plays a crucial role in academic stress. Teacher-related stress primarily includes academic expectations and the extent to which teachers recognize individual differences, but direct criticism has a stronger impact on stress levels. Peer stress originates from competition and social comparison, especially in highly competitive academic environments, where students may experience additional stress due to peer evaluation, as reflected in the item “When I fail, others mock me.”

This study systematically evaluated the reliability and validity of the scale and obtained favorable measurement results. First, Expert evaluation confirmed high content validity, with I-CVI meeting standards, S-CVI at a high level, and most K values excellent (Lynn, 1986). Previous studies lacked theory basis and content validity assessment (Ang and Huan, 2006; Sun et al., 2011), leading to potential item bias or omission of key stress sources. Our study fills this gap, confirming that the scale effectively measures academic stress across dimensions and is suitable for research and practice. Second, both EFA and CFA demonstrated the stability of the scale's structure. The factor structure extracted from EFA largely aligned with theoretical expectations. CFA fit indices met acceptable standards. The four-factor structure was generally consistent with theoretical constructs, though some details differed. For example, the cross-loading of Item 16 might indicate that it measures multiple constructs. Although this item showed cross-loading between the peer and self dimensions, this overlap is theoretically justified. According to the social comparison theory (Buunk and Gibbons, 2007) and achievement goal theory (Dweck and Leggett, 1988), the desire to outperform peers reflects both external comparative motivation (peer-related competition) and internalized self-evaluation (self-imposed standards). In academic settings, peer competition often becomes internalized as part of one's self-expectation to achieve or maintain superiority (Jansen et al., 2022), and it serves as an important source of academic stress (Sui, 2022). Therefore, this item naturally contains both peer-related stress and self-imposed stress. Next, this study confirmed measurement invariance across gender and regional groups, ensuring the scale's consistency. Previous studies lacked large samples to test regional measurement invariance (Ang and Huan, 2006; Sun et al., 2011). This study fills the gap, confirming the scale's equivalence across regions and ensuring reliable cross-regional research. Finally, the full-scale internal consistency reliability was good. The parent and self-imposed stress dimensions exhibited higher reliability, while the peer and teacher stress dimensions had relatively lower but still acceptable reliability.

This study examined the relationship between adolescent academic stress and emotions, validating the criterion-related evidence of the scale, supporting its reliability and validity. First, the study found that academic stress was positively correlated with anxiety and negatively correlated with hope, with a stronger correlation between academic stress and anxiety. This finding aligns with existing research (Gao, 2022). A possible explanation is that anxiety, as a high-arousal negative emotion, is more significantly affected by academic stress, whereas hope, a low-arousal positive emotion, is less influenced. Second, the study found that self-imposed stress had a stronger negative correlation with hope than overall academic stress. This result suggests that compared to external stressors (such as parent, teacher, and peer stress), adolescents' own expectations and self-evaluations have a greater impact on their sense of hope. A possible explanation is that self-imposed stress directly influences goal-setting and self-identity; when self-imposed stress is too high, individuals are more likely to lose confidence in the future, thereby diminishing their sense of hope (Cao and Ma, 2024). Additionally, the study analyzed the effects of different sources of stress. The results indicated that self-imposed stress had the most significant impact on hope, while parent, peer, and teacher stress had relatively smaller effects. This differs from some studies that suggest parent stress is the primary source of academic stress (Ang and Huan, 2006), possibly reflecting cultural differences in stress sources. In a culture that highly values academic achievement, adolescents may focus more on their own academic success rather than external expectations, leading self-imposed stress to become the primary influencing factor.

The findings of this study suggest that academic stress follows a normal distribution, which is consistent with common patterns in psychological measurement. Previous research has shown that academic stress can typically be divided into different levels, with moderate stress improving learning efficiency, while excessive or insufficient stress may impair academic performance or mental health (Ma, 2023). This study further determined specific academic stress level ranges, providing empirical support for educators and students in managing academic stress. Based on ROC analysis, two optimal cut-off points were identified, leading to the classification of low, moderate, and high academic stress levels (0-36 points for low stress, 37-46 points for moderate stress, and 47-84 points for excessive stress). The risk of anxiety disorder for high stress group is far higher than the moderate stress group, while the risk of moderate stress group is slightly higher than the low stress group. Additionally, the cut-off points were established based on the Youden index, optimizing sensitivity and specificity to ensure the scale's accuracy and practical usability in classification (Hajian-Tilaki, 2018).

This study employed network analysis and IRT to simplify the Academic Stressor Scale, selecting six core items. The results indicated that the key contributors to academic stress primarily stem from family, self, and teacher dimensions, with “My parents blame me,” “I am dissatisfied with my academic performance,” and “My teacher criticizes me” being the most significant factors. This finding highlights the crucial roles that family and school environments play in the formation of academic stress. The results align with previous research, which suggests that high parent expectations and negative feedback often exacerbate academic stress, potentially leading to anxiety or depression (Ang and Huan, 2006). Additionally, teachers' evaluation methods can impact students' academic self-concept, thereby influencing their motivation to learn (Hattie and Timperley, 2007). This study further confirmed the central role of these factors in the academic stress network through network analysis and validated item importance using IRT, supporting previous research on sources of academic stress. Furthermore, the study found that the centrality of academic stressors remained relatively stable across genders and regions, suggesting that these stressors may have strong generalizability rather than being exclusive to specific groups. The short form Academic Stressors Scale used the six core items (Items 3, 4, 6, 9, 13, and 19) to cover the major stressors of students. It is suggested that participants could use the short form as a reliable and valid alternate when the time and resources are limited.

Although the academic stressor scale and its simplified version have good reliability and validity and can be used for assessment and intervention, they still have several limitations. First, as a cross-sectional study, it cannot capture changes in academic stress levels over time, and future longitudinal research is needed to examine test-retest reliability and dynamic changes in academic stress. Second, potential sampling bias should be acknowledged. Data were collected through a voluntary mental health application, which may introduce self-selection bias and limit the representativeness of the sample. Such sampling limitations may affect the robustness of the evidence for gender measurement invariance and the establishment of ASS norms. In addition, the sample was restricted to a Chinese population, which may further constrain the generalizability of the findings to other cultural contexts. Future research should employ more representative sampling strategies and examine the cross-cultural applicability of the scale across diverse populations. Third, this study relied on self-reported questionnaires, which may have been influenced by social desirability bias. Future research could incorporate additional assessment methods, such as peer or teacher evaluations, to further validate the scale's effectiveness. A further limitation of this study is the absence of qualitative interviews or focus groups with students, which could have offered deeper insights into their perceptions of academic stressors. Future research should utilize the mixed-methods to strengthen the representativeness and depth of the scale development process. The resulting insights will inform the revision and refinement of the corresponding subscale items, thereby enhancing their reliability and validity.

## Data Availability

The raw data supporting the conclusions of this article will be made available by the authors, without undue reservation.
