# Supplementary material for: Development and validation of the academic stressors scale and its short version
Source: Front Psychol. 2026 May 21;17:1790872. doi: 10.3389/fpsyg.2026.1790872 (PMC13233693; doi:10.3389/fpsyg.2026.1790872)
Supplement: Supplementary file 1 [file Supplementary_file_1.docx]

### Supplemental Material

Section 1. Exploratory factor analysis.

Section 2. Criterion-related validity.

Section 3. The normal distribution of academic stress.

Section 4. ROC Curve.

Section 5. Accuracy and stability of the network.

Section 6. Item information curves.

### Section 1. Exploratory factor analysis.


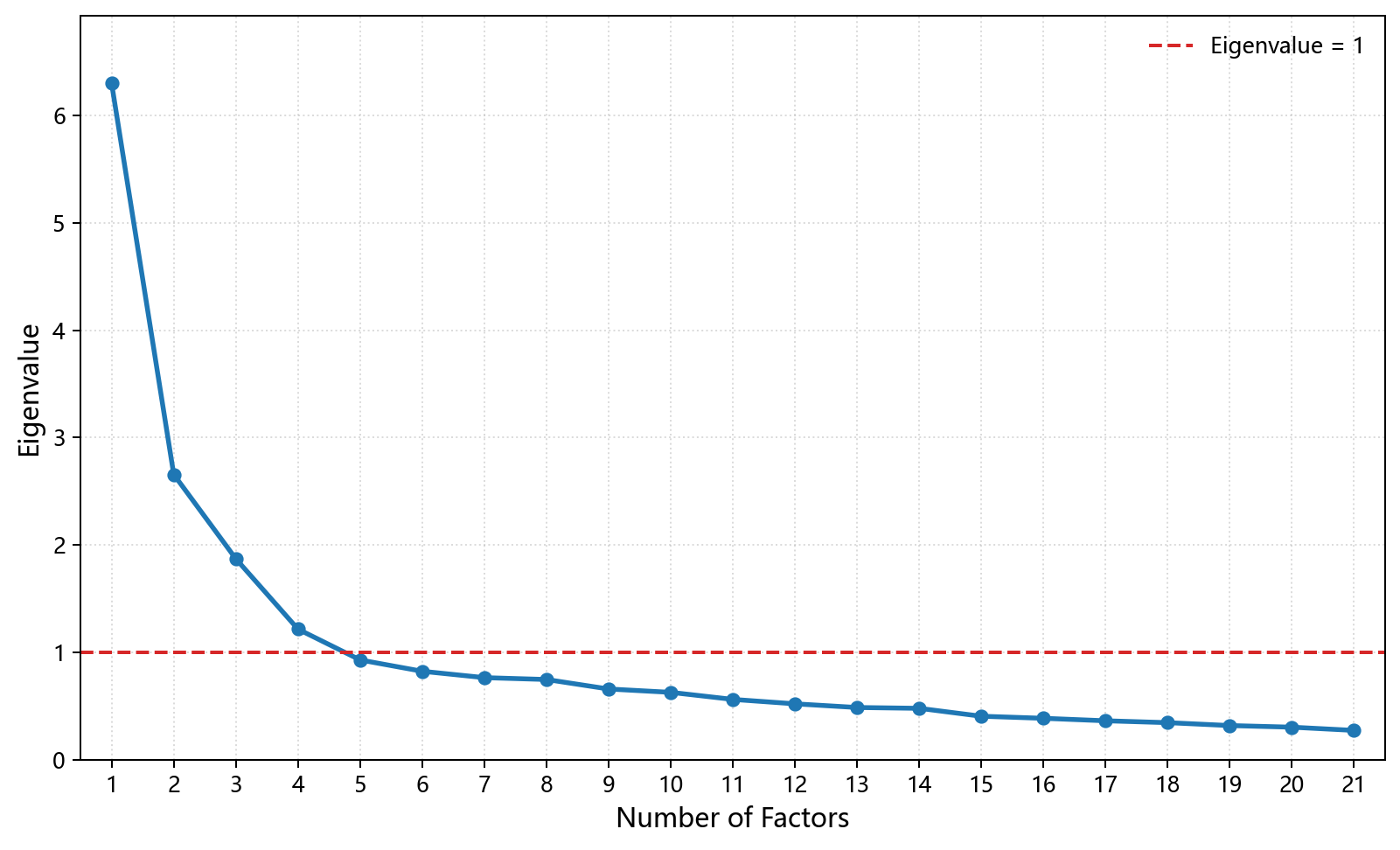


Figure S1. Scree plot.

### Section 2. Criterion-related validity.


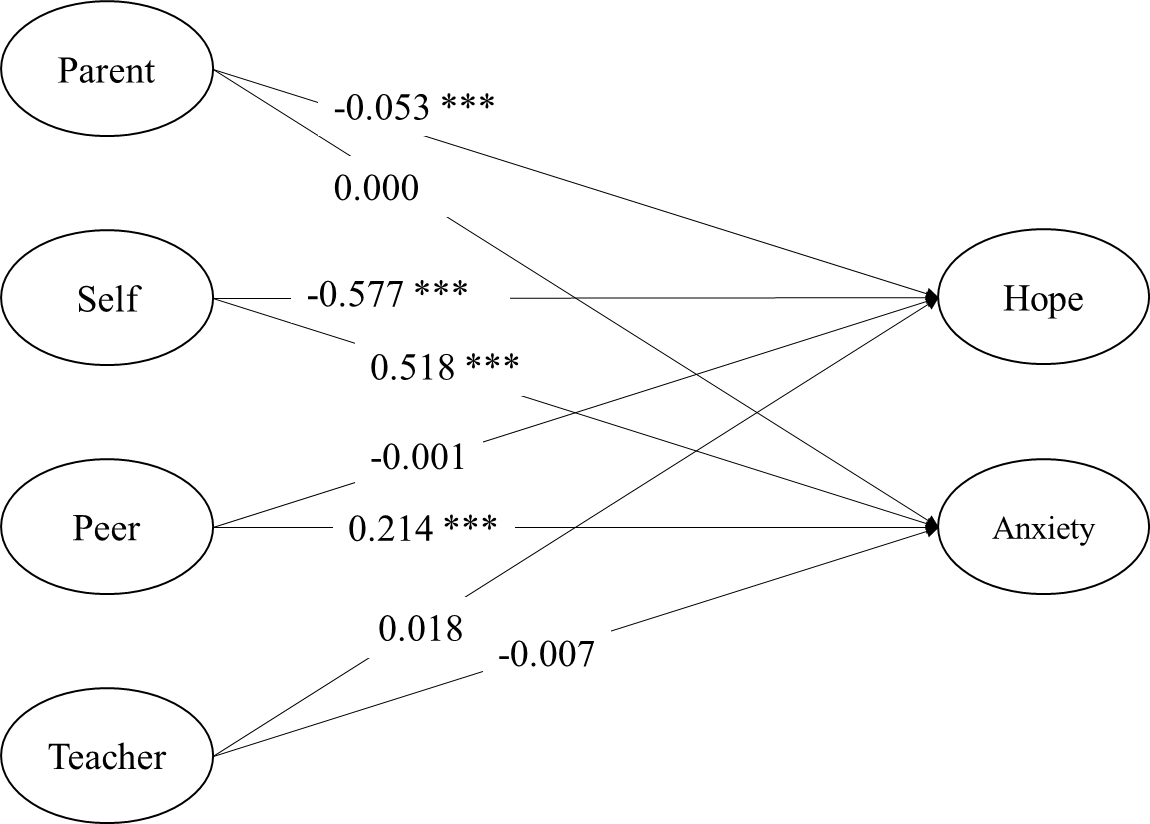


Figure S2. Structural equation model summarizing the relationships between the four dimensions of academic stress and the outcome variables of hope and anxiety.

****p* < 0.001.

### Section 3. The normal distribution of academic stress.


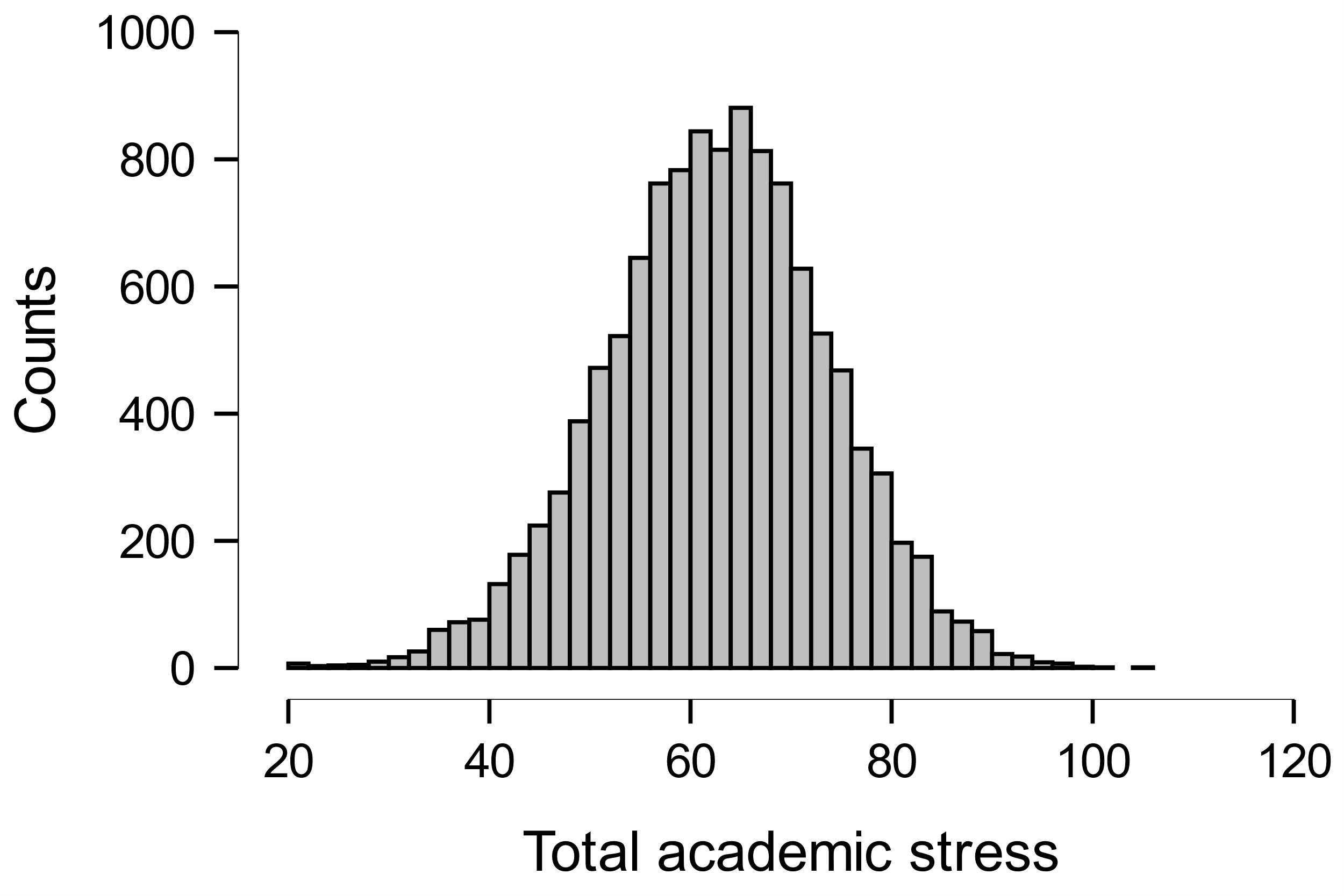


Figure S3. Frequency histogram of total academic stress.

Figure S4. Q-Q plot of total academic stress.

### Section 4. ROC Curve.


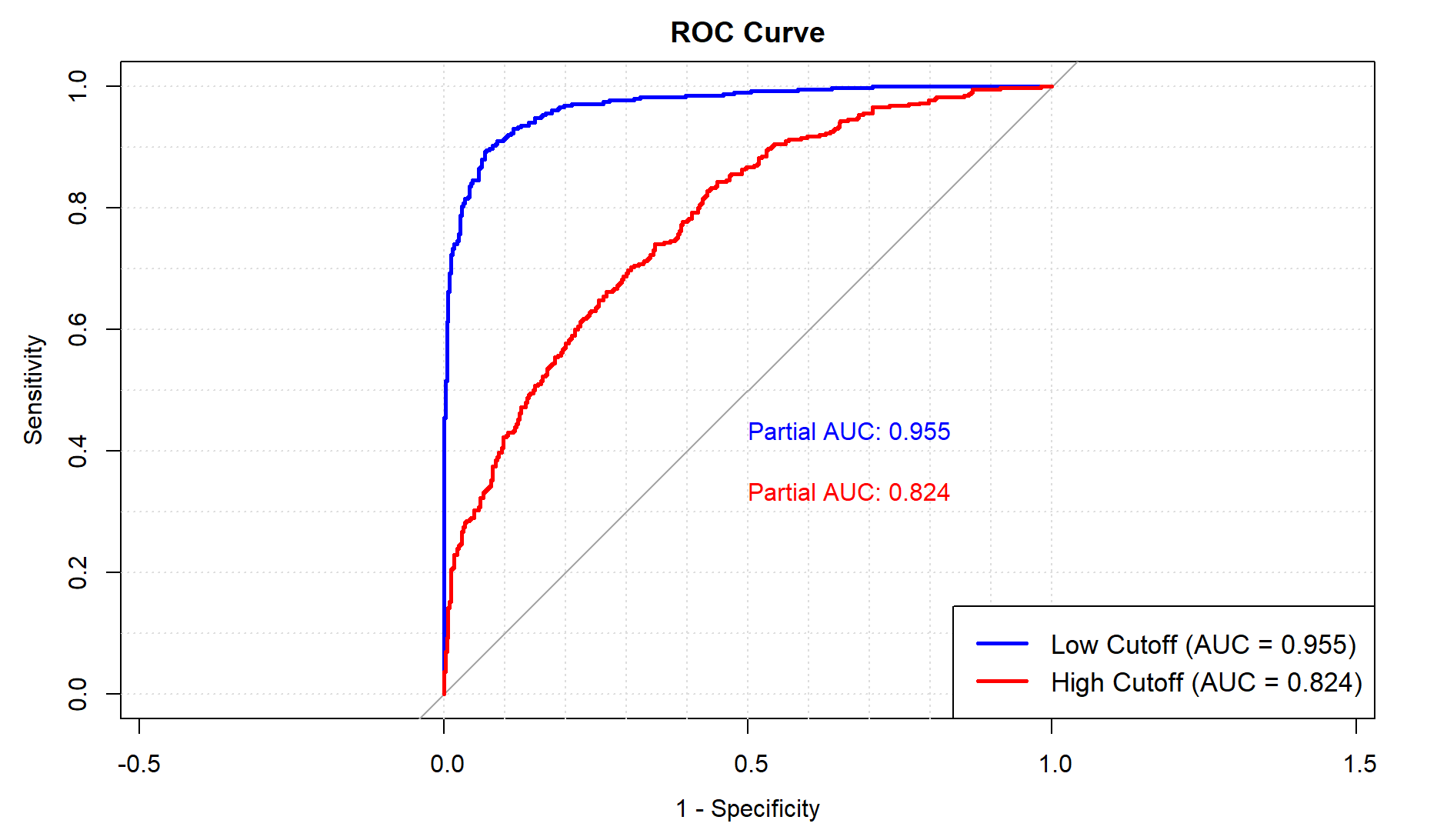


Figure S5. ROC Curve.

### Section 5. Accuracy and stability of the network.

Figure S6. Edge-weight accuracy Bootstrapped confidence intervals of estimated edge-weights for the estimated network. The red line indicates the sample values and the gray area indicates the bootstrapped confidence intervals. Each horizontal line represents one edge of the network, ordered from the edge with the highest edge-weight to the edge with the lowest edge-weight.

Figure S7. Stability of node expected influences. The x-axis illustrates the sample decrease from 95% to 25% of the original sample, and the y-axis illustrates the changes in correlation estimates between the subsample and the original entire sample. Lines indicate the means, and areas indicate the range from the 2.5th quantile to the 97.5th quantile.

### Section 6. Item information curves.


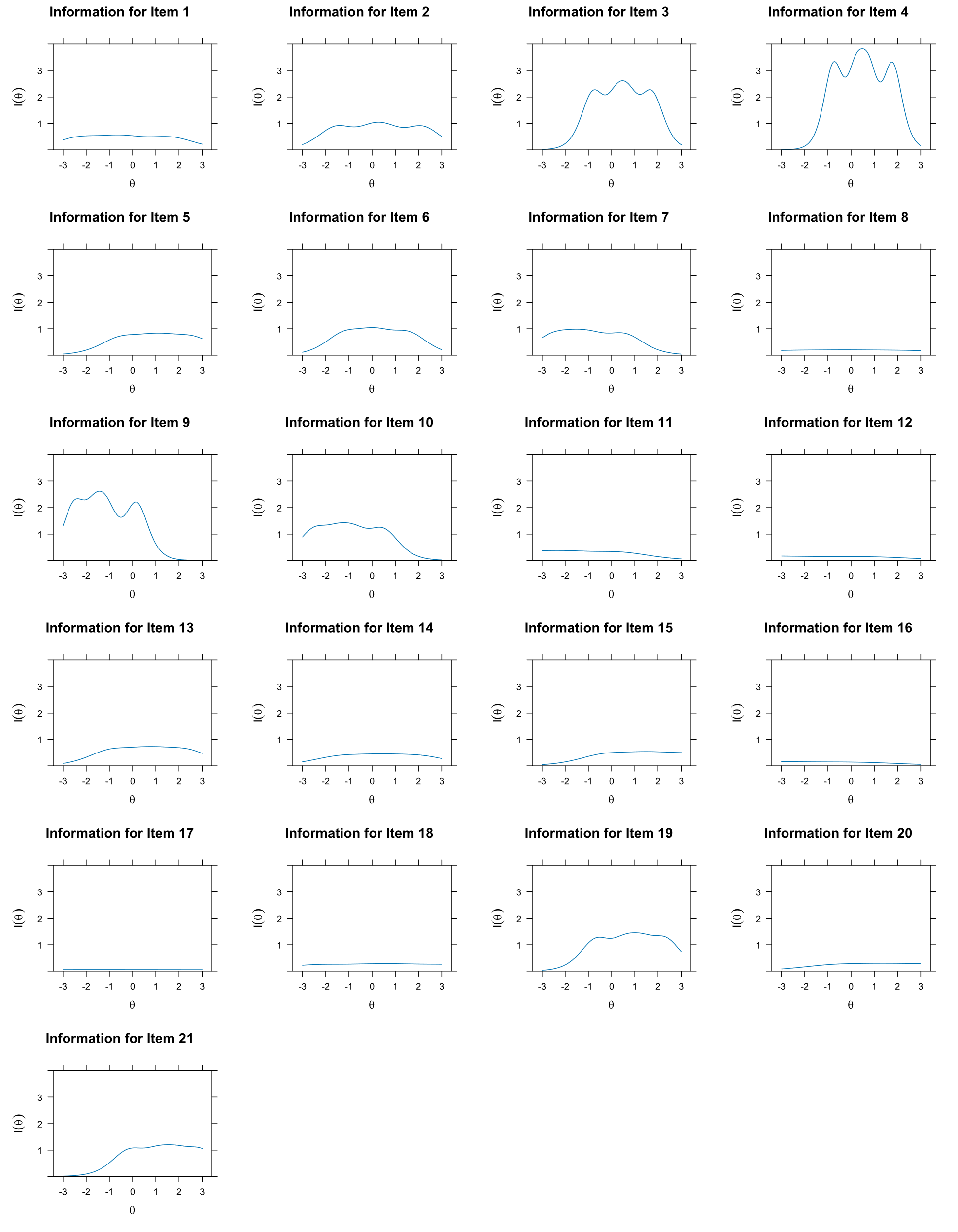


Figure S8. Item information curves of each item.
